# Accessibility, Agency, and Trust: A Study About Equestrians' (Online) Learning Repertoires

**DOI:** 10.3389/fspor.2022.863014

**Published:** 2022-04-28

**Authors:** Lovisa Broms, Klara Boije af Gennäs, Aage Radmann, Susanna Hedenborg

**Affiliations:** ^1^Department of Sport Science, Malmö University, Malmö, Sweden; ^2^Department of Teacher Education and Outdoor Life Studies, Norwegian School of Sport Sciences, Oslo, Norway

**Keywords:** equestrian sport, ICTs, online repertoires, learning repertoires, social media, horse-knowledge

## Abstract

Todays' online media landscape facilitates communication on how sports practitioners can develop in their sport. Hence, sports and educational institutions need to recognize the increased role of the individual as “a facilitator of knowledge” through *information and communications technology* (ICT). For sport organizations and educational institutions to effectively reach out with knowledge and research, they need to know how individuals assess, value, and trust information sources. This article aims to increase the knowledge and understanding of how the traditional culture in equestrianism meets the contemporary media user. It is based on a study that uses a mixed methods design, containing a questionnaire with 1,655 respondents and 28 focus group interviews with Swedish and Norwegian equestrians, to investigate how equestrians create their own repertoires of horse-knowledge online and what sources of knowledge they trust and prioritize. The results show that accessibility, agency, and trust are key terms when mapping equestrians' preferred knowledge platforms, and that equestrians are generally not satisfied with the availability and the quality of horse-related online content. Horse experience is the most important positional factor influencing online repertoires in the equestrian community. Riders with less experience turn to Social Network Sites (SNS) to a higher extent than riders with more experience. Further, equestrians find the ability to assess information as an important yet challenging task. This article shows that the term *(online) learning repertoires* is appropriate when discussing the relationship (or *clash*) between the traditional culture in equestrian sports and the contemporary media user. On the one hand, many equestrians clearly express that they would rather stay away from obtaining information about horses and riding on ICTs. On the other hand, the data, together with previous research, indicates that many equestrians see ICTs as important platforms for discussing and exchanging information about horses and riding.

## Introduction

There is a growing body of research suggesting that the traditional sports and physical activity sector would benefit from adopting, or getting inspired by, the new, digitalized way of exchanging knowledge seen in self-organized lifestyle sports (Jones, 2011; Enright and Gard, 2016; Säfvenbom et al., 2018; Säfvenbom and Stjernvang, 2019). However, what happens when practitioners in traditional sports “go online” to obtain and exchange knowledge? Equestrian sports are among the most popular children and youth sports in Sweden and Norway. Most of the members in the Swedish and Norwegian Equestrian Federations are girls and women (Hedenborg et al., 2021). Previous research on the history of equestrian sports shows that equestrianism is a traditional sport where a military heritage is seen in the stables and in its learning cultures and where institutionalized governance structures lead to slow development pathways (Cf. Thorell and Hedenborg, 2015). However, in parallel with societal and technological developments, there have been changes in how equestrians search for information about horses and riding. Several studies show that equestrians, just like practitioners in self-organized lifestyle sports, use new media to obtain and exchange knowledge (Bolwell et al., 2013; Byström et al., 2015; Lofgren et al., 2016; Dashper, 2017; Hii et al., 2020; Broms et al., 2021). This development in equestrian sports may have led to a change in ideas on formal and informal education and learning in the stable and in the equestrian ring. In parallel, Swedish insurance companies see an increased number of cases where horse owners show a lack of care by not calling the veterinarian when the horse is injured or sick (Grundberg, 2020). We believe that one explanation for this tendency toward such lack of care could be the *increased accessibility* of horse-related information through information and communications technologies (ICTs). Therefore, it would be of interest to investigate whether knowledge exchange through ICTs has already replaced or complemented the more traditional means of knowledge exchange, or if this is even possible. An important question is whether traditional sources of knowledge—such as the practical learning in the stable environment (Greiff and Hedenborg, 2007; Hedenborg, 2008), educational materials (e.g., *Arméns ridlära* and *Lilla Ridboken*) (Hedenborg, 2009), and institutional education—have been exchanged for quickly obtainable knowledge through ICTs and, if so, to what extent.

Recent research show that Swedish and Norwegian equestrians generally are comfortable with their own ability to critically assess information about horses and riding distributed on ICTs. However, many respondents express a *lack of trust* toward other, less experienced, equestrians' abilities to use ICTs as knowledge platforms (Broms et al., 2021). The dichotomy of riders' confidence of their own experience, their lack of trust toward other riders' abilities, and the quickly developing digital landscapes is a particularly interesting case. We argue that it is important to further investigate the use of ICTs as knowledge platforms from the perspective of equestrians to create an increased understanding of the risks and benefits with the developing digital media landscapes. Therefore, this article aims to generate increased knowledge and understanding of how the traditional culture in equestrianism meets the contemporary media user and to discuss what this means for the developments of online repertoires within sports. We are interested in, from a media theoretically informed perspective, analyzing in what way equestrians create their own repertoires of horse-knowledge and what sources of knowledge they trust and prioritize.

First, we will create an increased understanding of equestrians as media users by using the concept of *online repertoires*. Online repertoires is a concept that enables an understanding of people's online habits. It refers to what users do when they are online, and it includes all types of online platforms and devices (Olsson et al., 2019). Second, we will discuss the type of sources of knowledge that equestrians prioritize and trust. Research indicates that trust in institutions, specifically media, is decreasing today (Kavanagh and Rich, 2018), which increases the need to constantly and critically assess information and its sources (Haider and Sundin, 2020). Haider and Sundin (2020) argue that there is a need to understand this process of assessment. It is important for stakeholders within the equestrian community (e.g., national federations, insurance companies, veterinarians, riding schools, etc.) to understand what knowledge equestrians and horse owners obtain through social media and the internet and, more importantly, how they value this information. Contemporary media landscapes imply major changes for sporting communities. Everyone, from the individual practitioner to large sports bodies, is affected in some way. Although the current study specifically focuses on equestrian communities in Sweden and Norway, the thematic analysis and discussion on online repertoires, trust, and the ways ICTs are used for learning and development are indeed relevant for the larger sports context. The welfare of horses is a top priority in equestrian sports, and as mentioned above, there are indications of an increasing number of cases involving a lack of care (for the horses). We argue that it is important to understand and embrace the contemporary media landscapes, but in parallel, we must also understand how individuals search for and value information communicated through ICTs. If we do not know how individuals assess, value, and trust sources of information, it may be challenging for organizations and educational institutions to reach out with evidence-based knowledge and research.

The following research questions guide our study:

1. What defines equestrians' online repertoires?
2. How do equestrians assess and value information on horses and riding collected through ICTs, what sources do they trust, and why do they trust these sources?
3. What is the relationship between horse-related experience, age, and equestrians' use of ICTs as information platforms?

## Background and Theoretical Frame

### Online Repertoires

To increase understanding of contemporary media users, Uwe Hasebrink has developed a repertoire-oriented approach focusing on how different media platforms and different kinds of content are combined by the user. He uses the notion of *media repertoires* to understand results of different forms of *selectivity* (Hasebrink and Popp, 2006; cf. Hasebrink and Domeyer, 2012; Hasebrink and Hepp, 2017). Media repertoires can be understood as “outcomes of *structural, positional*, and *individual* factors” (Olsson et al., 2019: 40–41). Structural factors include the types and the quality of media available to specific users, whereas positional factors involve “users' positions within a social structure” (i.e., gender, age, degree of education, etc.) and how these positions influence everyday media repertoires. Individual factors concern the variations in media repertoires within both structural and positional factors. Particularly, they estimate the impact of individual preference within the aforementioned factors. There are three distinct principles to consider when using media repertoires as an analytical tool. The *user-centered perspective* focuses on the user instead of the media. *Entirety* relates to the importance of considering the whole variety of media that a person assembles (Hasebrink and Domeyer, 2012; Hasebrink and Hepp, 2017; Olsson et al., 2019). *Relationality* refers to “how users intermingle different media assets and turn them into a coherent whole” (Olsson et al., 2019:41).

For this study, we decided to limit the concept of media repertoires to *online repertoires*, which refers to what users do when they interact on the internet (Olsson et al., 2019). The concept of *online repertoires* enables researchers to capture the diversity of online tools offered to users and works as an analytical framework to organize current online options offered to users online (Olsson et al., 2019). Moreover, online repertoires are not a set phenomenon. The concept follows the continuously evolving digital landscape. Thus, it is suitable for our study to observe what equestrians do when they interact on the internet and how they value information obtained through online sources about horses and riding. To get a more extensive understanding of equestrians' repertoires in relation to the exchange of horse-knowledge online, we focus on the use of not only social media, but all kinds of online platforms. Therefore, we use the term Information and Communications Technology (ICT). Specifically, we focus on how equestrians discuss critical assessment and, more importantly, how they describe their own process of selecting and assessing information about horses and riding obtained through ICTs.

### Imagined Affordances

The cognitive psychologist Gibson (1979) developed the term *affordances*, which has become a commonly used notion in communication technology and social media studies (Nagy and Neff, 2015; Shaw, 2017; Manzerolle and Daubs, 2021). In broad terms, affordance is what the environment offers the animal. In his book *The Ecological Approach to Visual Perception* (1979), Gibson defines it as follows:

The *affordances* of the environment are what it *offers* the animal, what it *provides* or *furnishes*, either good or ill. The verb *to afford* is found in the dictionary, the noun *affordance* is not. I have made it up. I mean by it something that refers to both the environment and the animal in a way that no existing term does. It implies the complementary of the animal and the environment. (Gibson, 1979:127)

Shaw (2017) states that one can think of interactive media technologies in terms of imagined affordances and raises Nagy and Neff's (2015) suggestion that an extension of the affordance theory can support communication theorists to focus on the construction, mediation, and materialization of power and social relationships. Nagy and Neff (2015) further suggest that *imagined affordance* helps to explain how people shape their media environments, perceive them, and have agency within them because of imagined affordances. In this article, we will use the term imagined affordances to further develop the analysis around online repertoires in relation to equestrians' knowledge exchange through ICTs.

### Equestrians' Online Repertoires

There are several studies on ICTs in relation to equestrianism. For instance, Dashper (2017) analyzes the content of blogs by British amateur and professional riders. This study shows that ICTs allow equestrians to exchange stories about relationships between horses and humans, and that these platforms have developed as important spaces for creating norms for horse keeping. Dashper (2017) emphasizes that ICTs have become important recourses for equestrians who, through these platforms, can stay up to date on what is going on in the equestrian world. However, she argues that there are issues with, for example, the spread of false information, and that this type of information can make horse owners doubt and, in the worst-case, refrain from taking veterinarians' advice (Dashper, 2017). Inspired by the autoethnographic method, Dashper (2017) reflects on how she herself, despite the veterinarian's advice, uses Google to search for information about her horse's injury. In a study on Information seeking behavior among members of the American horse competition industry, Lofgren et al. (2016) show that 86% of the respondents used the internet as a horse-health information source. However, they did not see the internet as a preferred horse-health information source. Instead, they preferred veterinarians (91%), farriers (77%), and trainers (68%) (Lofgren et al., 2016). Similar results are seen in a study aiming to create understanding of where UK leisure horse owners seek advice and information on four subject areas relevant to horse welfare: horse behavior, health, stable care, and training. This study shows that internet/forums were the third to the fifth most used of the seven information sources (Hockenhull and Creighton, 2013). In another study, aiming to identify the research-based educational needs and preferred dissemination methods within the Thoroughbred and Standardbred racing and breeding industries in New Zealand, Bolwell et al. (2013) show that veterinarians, websites, friends or other horse owners, and printed magazines were used most often (and were most preferred) for equine research. Social media were the least used and preferred sources of information. However, Bolwell et al. (2013) argue that this is likely due to the age demographics of the study, where most of the respondents who did prefer social media were younger (below 40 years old).

Interestingly, recent research by Broms et al. (2021) shows that riders in all age groups in Norway and Sweden retrieve information, knowledge, and inspiration about horses and riding on Social Network Sites (SNS). Facebook is the most popular SNS for the older generation, whereas Instagram and Snapchat are more commonly used by the younger generation. However, these respondents use SNS more commonly to obtain knowledge about horses in general rather than to seek specific information about, for example, training or diseases and injuries. On the other hand, the riders in Broms et al.'s (2021) study state that other riders use social media to learn how to take care of injured horses, and they stress that riders with less experience than themselves lack insights on how to use social media to obtain information about horses and riding in an informed way. Meanwhile, Hii et al. (2020) suggest that a combination of traditional and new information channels is required to reach different audiences with horse-health information. Another study shows that intimacy and the common love for horses creates intimate bonds between equestrian influencers and their followers. By sharing pictures, videos, and comments on their everyday lives, equestrian influencers produce knowledge about equestrianism and share information on how horses should be cared for (Radmann et al., 2021). Byström et al. (2015) analyze how safety aspects are communicated by Swedish equestrians on ICTs. This study shows that safety is a rarely discussed topic and that, when it is discussed, it is mainly the horses' safety that is emphasized. Byström et al. (2015) argue that ICTs are both risk factors and valuable tools in the quest to increase safety measures within the equestrian community. In sum, previous research on equestrians' information seeking behavior indicate that equestrians in different countries who are involved in different areas of the equine sector generally do use ICTs as information sources on horses and riding, but they seem to prefer other information sources. Most of the abovementioned studies were published more than 5 years ago and indicate that younger equestrians seem to be keener on and are more comfortable with using ICTs and express that there is a need to further investigate future possibilities of reaching out with horse-related information through ICTs. Thus, the present study will contribute to the field by generating increased knowledge and understanding of how the traditional culture in equestrianism meets the contemporary media user.

### Critical Information Assessment Behavior

Using online communities to satisfy information needs is becoming more common, and there are numerous ways to seek information online (Shah, 2017). The concept *social search* exists at the intersection of information seeking and social media. It refers to two aspects: people looking for information that is socially constructed and people using social connections to look for information. Social search on ICTs has proven to be a popular method for seeking information (Shah, 2017). As a result, a growing body of research highlighting the importance of critical evaluation of information and critical assessment of information sources has emerged.

**Figure 1:**
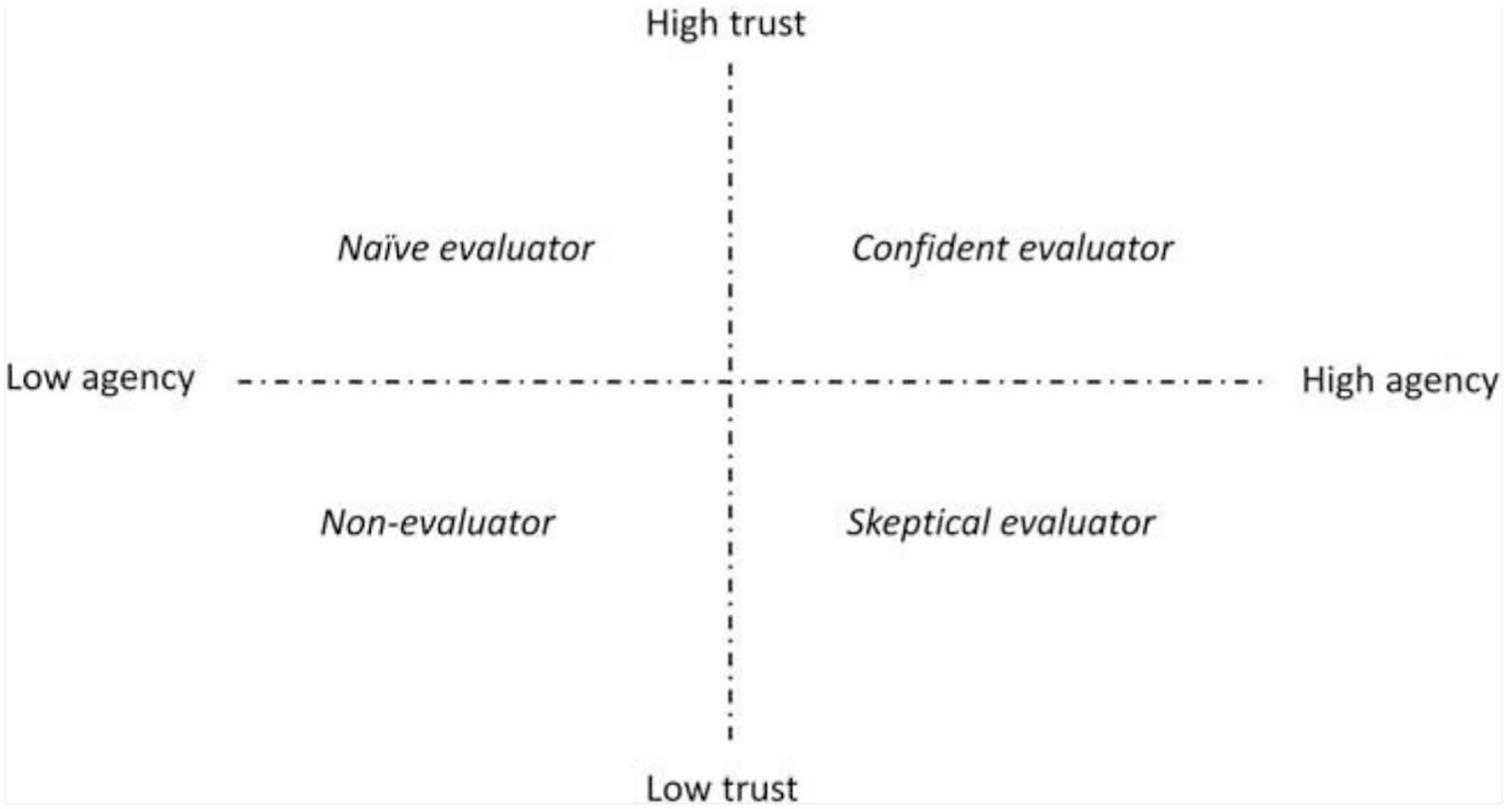
Information assessment stereotypes matrix (Haider and Sundin, 2020: 12).

Relating to critical information assessment behavior, there is concurrently a shift from mistrust toward distrust. That is, the question changes from “how is this true to how is this false” (Haider and Sundin, 2020:3). Trust is a crucial aspect for understanding critical assessment of credibility, but it concerns trust in institutions, methods, and systems rather than in individuals (Haider and Sundin, 2020:14; Hardin, 2002). The *information assessment stereotypes matrix* (Figure 1) developed by Haider and Sundin (2020) allows us to use trust and individual agency as dimensions to map out the critical assessment of information among equestrians. It allows us to understand equestrians' online repertoires and increase the knowledge on how equestrians trust and value information about horses and riding.

The *naïve evaluator* is defined by a combination of low agency and high trust. For example, naïve evaluators tend to believe in what they find without considering themselves as actively involved in making that judgement. This behavior is encouraged by providers of algorithmically curated content. Alongside *non-evaluators*, who are defined by low agency and low trust, *naïve evaluators* are often used as so-called *strawmen*, embodying “the other.” Respondents in Haider and Sundin's (2020) study use the notions of *naïve-* and *non-evaluators* as persons to keep distance from and persons to compare oneself with. On the other hand, the *skeptical evaluator* is defined by low trust and high agency. Persons in this group do not really trust anything they encounter, but they are simultaneously very invested in evaluating information themselves. Lastly, *confident evaluators* consider themselves capable of evaluating information, and they put their trust in authoritative information.

### Dunning-Kruger Effect

Another useful perspective for this article is the *Dunning-Kruger effect*. Coined by Kruger and Dunning (1999), this term explains how people tend to “hold overly favorable views of their abilities in many social and intellectual domains” (Kruger and Dunning, 1999:1121). Their study reveals that this overestimation generally appears when people with low ability for a task overrate their own ability and, on the contrary, people with high ability for a task generally underestimate their own ability. In a study on public opinion about vaccination policies, Motta et al. (2018) argue that the Dunning-Kruger effect can help explain public opposition to vaccination policies. More than a third of the respondents in their study thought that they knew as much or more than doctors and scientists about the cause of autism (Motta et al., 2018).

## Methodology

### Design

In this study, we used an exploratory sequential mixed methods design (Creswell and Creswell, 2018). We adopted the mixed method definition used by Tashakkori and Teddlie (2003). The idea of mixed methods is that by collecting both qualitative and quantitative data, we can neutralize their weaknesses (Creswell and Creswell, 2018) and enhance the understanding of a phenomenon (Gratton and Jones, 2004). The project first began with a qualitative phase of focus group interviews. This was followed by a quantitative phase with a questionnaire built on the participants' views from the first phase and, finally, additional focus group interviews. The design could be described as QUAL → quan (Tashakkori and Teddlie, 2003). The sample scheme was simple, which means that the desired population (i.e., equestrians) had an equal and independent chance of participating in the study (Tashakkori and Teddlie, 2003; Creswell and Creswell, 2018). Further, we kept in mind that the triangulation of data could support the researchers to describe the phenomenon in greater detail. To maintain transparency of the triangulation, we thoroughly outlined the data collection and data analysis (Pluye et al., 2018) below.

### Data Collection and Sample Selection

The first round of qualitative data was collected through focus group interviews conducted between March and June 2017, wherein we interviewed equestrians studying at upper secondary schools with an equestrian sports profile in Sweden (i.e., equestrians in the same age group with similar experiences with horses and riding). After completing the first round of qualitative data collection, we analyzed and discussed the material and preliminary findings. As the first round of interviews were conducted with a rather homogenous group of equestrians, the initial analysis indicated a clear need for posing questions to a broader population of equestrians with different levels of experience and in different age groups. To achieve this, we constructed a questionnaire and conducted a quantitative study in the second quantitative phase.

The questionnaire was constructed to expand the qualitative findings and the knowledge of social media habits among a broader group of equestrians. A post with a link to the online questionnaire was available on the Facebook page of the research project from 7 May 2018 to 30 September 2018. The questionnaire was also shared by the Swedish and Norwegian Equestrian Federations through their social media accounts. In addition, it was disseminated with the help of the Swedish equestrian centers Ridskolan Strömsholm and Flyinge AB. This manner of dissemination and convenience-sampling allowed us to reach a large group of people. In the third and last phase of data collection, we conducted more focus group interviews to deepen our understanding of how equestrians use ICTs.

The second set of interviews and third phase of the data collection were conducted between August and December 2018. The interviewees were equestrians studying at the upper secondary level in Sweden and Norway (*n* = 39), students at riding schools in Sweden and Norway (*n* = 31), and horse owners in Sweden and Norway (*n* = 33).

### Focus Group Interviews

The interview material consists of 12 semi-structured focus group interviews in Norway (*n* = 47) and 16 focus group interviews in Sweden (*n* = 56) (2–6 participants in each group). The interviewees were recruited by contacting gatekeepers at upper secondary schools with an equestrian sports profile, riding schools, and private stables. The gatekeepers identified interested individuals who were willing to partake in the interviews. All interviewees were thoroughly informed about the aim and nature of the study and signed an informed consent form. The interviews lasted between 20 and 60 min (average duration of 56 min) and were of a semi-structured character. In focus group interviews, the researcher assembles a group of people who were to discuss a given subject (Wibeck, 2010). The word *focus* indicates that the discussion centers on a predetermined topic. In our study, we constructed an interview guide to ensure a focused discussion on the research topic. However, the interviewees discussed the topics of the guide freely and had plenty of room to elaborate on the topics without the influence of the researcher.

The interview guide consisted of questions in three categories. The first category included open introductory questions relating to how the interviewees were engaged with horses and riding, why they chose to engage in equestrian sports, and whether they had any equestrian role model(s). The second category comprised questions about the interviewees' habits in relation to social media, namely, what type of platforms they used; how much time they spent on these platforms; and what content relating to equestrianism they generally read, engaged with, and created on these platforms. Finally, there were questions regarding knowledge, social media, and equestrianism. The interviewees discussed whether they believe that one could gather knowledge about horses and riding through social media, whether they used social media to retrieve knowledge about horses and riding, and what type of content they collected and produced. The interview guide was tested in a focus group interview and was evaluated afterward by the research group. No revisions were needed, and the same guide was used for the additional interviews.

Analyzing focus group interviews primarily entails coding the material, dividing it into units, and searching for trends and patterns (Wibeck, 2010). Thus, the interviews were recorded, transcribed, and subjected to thematic analysis (Clarke and Braun, 2018) using the qualitative data analysis program Nvivo12. During the transcription process, we pseudonymized the interviewees. The transcripts were read and re-read several times by two of the authors.

### Questionnaire

The online questionnaire consisted of 44 questions aiming to investigate the way in which equestrians in Sweden and Norway use ICTs in relation to their interest in horses and riding. In this article, we used 10 questions of relevance for the aim of the article. The first set of questions was designed to inform us about the participants' background, including gender, age, and country. The second part was designed to establish the participants' experience in equestrian sport, and the third part was designed to explore equestrians' use of ICTs in general and in relation to horses. The inclusion criteria consisted of respondents ≥ 16 years of age living in Sweden or Norway. The exclusion criteria consisted of respondents failing to report age, gender, and country.

First, we performed a descriptive analysis using crosstabulation, followed by a correlation analysis to investigate the relationship between ICT use and age and also horse experience. Both age and horse experience were divided into three categories. For age, the categories were low = 15–18 years, medium = 19–30 years, and high > 30 years. Horse experience is defined by how many years of experience the respondents have with horses and riding, so the categories were low ≤ 5 years, medium = 6–16 years, and high > 16 years. The analysis was performed using IBM SPSS version 27.

We merged the responses to the following two open-ended questions to represent the type of ICTs equestrians use to find knowledge about horses: “What type of internet platforms do you use to find knowledge about horses?” and “What type of SNS do you use to find knowledge about horses?” The open-ended questions were then re-coded into nine manifest codes using thematic analysis (Clarke and Braun, 2018). The first four manifest codes were based on existing definitions of ICTs (Nicholas and Rowlands, 2011; McCay-Peet and Quan-Haase, 2017), namely, (1) social network sites (SNS), (2) media sharing, (3) blogs and forum, and (4) collaborative authoring. The five additional manifest codes were constructed and identified as (5) IRL (in real life), (6) official websites, (7) research, (8) commercial websites, and (9) search engines. We merged and analyzed the data using Microsoft Excel version 16.53. Table 1 below shows what types of sources guided the construction of themes.

**Table 1:** Number (and percentage of) users using different types of information and communications technologies (ICTs) to obtain information on horses and riding.

**Type of ICT**	N^*^(%)	**Examples of ICTs**
Search engines	487 (33.38)	Google, Safari
SNS	454 (31.12)	Facebook, Instagram, Snapchat
Commercial websites	344 (23.58)	Hippson, Agria
Official websites	253 (17.34)	ridsport.se, rytter.no
Research	207 (14.19)	SLU, Hästsverige.se, Jordbruksverket
Media sharing	151 (10.35)	YouTube, Podcasts
Blogs and forum	103 (7.06)	Bukefalos, Flashback
In real life	15 (1.03)	Asking a trainer or talking to a friend
Collaborative authoring	10 (0.69)	Wikipedia

* *N = 1,459. The respondents reported several ICTs; therefore, the total number of responses may exceed the total number of respondents (the proportion is calculated in relation to the total number of respondents)*.

Overall, 1,655 respondents replied, and after the exclusion process, 1,459 respondents remained. The sample had a mean age of 41.3 years (SD = ± 14.6), ranging between 16 and 77 years (95.9% respondents identified as female). A total of 1,170 lived in Sweden and 365 in Norway.

## Results

### Online Repertoires Among Equestrians

Initially, we asked the respondents in the questionnaire what type of ICTs they use to obtain information about horses and riding. On the question “Do you use SNS in general?,” 1,425 (97.7%) of the respondents answered “yes” and 34 (2.3%) answered “no.” They were also asked if they used SNS to find knowledge about horses, for which 688 (47.2%) answered “yes,” 549 (37.6%) answered “no,” and 222 (15.2%) failed to report. When asked if they use the internet to find knowledge about horses, 1,102 (75.5%) answered “yes,” 135 (9.3%) answered “no,” and 222 (15.2%) failed to report. In comparison to the study by Lofgren et al. (2016), where 86% of the respondents stated that they used the internet as a horse-health information source, fewer of the respondents in this study reported to use ICTs as knowledge platforms. We wanted to increase the understanding about equestrians' online repertoires and their use of different types of online platforms, not only their use of SNS. Therefore, we merged the responses to the two open-ended questions regarding (1) the type of SNS they use to obtain information about horses and riding (*n* = 615) and (2) the type of internet platforms they use to obtain information about horses and riding (*n* = 925). Table 1 (below) shows an overview of the results of the manifest coding from these two questions.

The overview of the ICTs used to obtain information about horses and riding shows that *search engines* (such as Google) are the most used ICT, *SNS* (such as Facebook and Instagram) come in second place, and the third most used ICT is *commercial websites*. Even though the questionnaire focused on the use of ICTs, a few (15) respondents chose to answer that they would rather acquire information about horses and riding from “live sources” such as their trainer or a friend in the stable.

Similar to the questionnaire, the focus group interviews show that many of the equestrians use ICTs, such as *search engines*, rather than *SNS*, for example, Facebook and Instagram, as a source of information on horses and riding. This is how a group of interviewees discusses whether they obtain knowledge about horses and riding through ICTs:

Researcher: Would you say that you can obtain knowledge about horses and riding by being active on the internet and social media?
R1: I'm not very active on social media, so I don't get very much from there. But I google a little here and a little there, but not that much information appears and it's very different information, because people have so many different views about horses and riding. So, I don't take it very seriously. I mostly listen to different experiences from people here at my stable.
R2: For me, it's the same, I don't google that much, I talk to the ones I know.
R3: I use the internet a lot and google a lot. I get a lot of information and have used the internet for many years and use sites that I know are serious. Of course, there are many forums where people express different viewpoints about lots of different things, so it's very important to use your common sense and “gut feeling.” I'm used to animals, and I'm brought up at a farm. So, I collect a lot of information from the internet, social media, and internet forums.

The three focus group participants quoted above emphasize that it is imperative to be aware that not everything, or even very little, of the information produced, shared, and discussed on ICTs can be trusted. A key feature in many of the focus group discussions around their online repertoires is that it could be risky and simply dumb to rely too much on what is written on, for example, *blogs and forums* and in Facebook groups. However, there are groups of interviewees who indicate that they do use ICTs as a source for knowledge and that this is indeed common among equestrians. In fact, many of the participants in the focus groups state that “other equestrians” use ICTs to obtain knowledge on horses and riding even if they do not do it themselves. When they confess that they do use ICTs, just like R3 expresses above, they find it important to mention that one needs to have a certain amount of experience with horses (i.e., agency) to be able to assess and use the information in a correct and safe way. Further, the equestrians express that they mostly listen to, and rely on, equestrians they know. For example, friends or coaches who they meet at the stable or at the riding school.

The equestrians seem to be hesitant to ask questions through, SNS but are more comfortable with asking search engines such as Google their horse-related questions. We argue that this might be because they are critical against the credibility of information on SNS. Another explanation could be that one does not want to be personally associated with the question, the answers, or the persons behind the answers. In this case, it is perhaps safer and more comfortable to ask Google, where the question will not be seen by anyone but the search engine (and the commercial interests behind it). This indicates that there are norms regarding how equestrians should, or perhaps rather should not, obtain information about horses and riding. Many of the participants in this study claim that they would rather not use ICTs at all to obtain information about horses and riding. Therefore, we argue that it is more appropriate to use the term *(online) learning repertoires*, with brackets around *online*, when discussing knowledge exchange among equestrians. The brackets indicate that equestrians' learning repertoires only to some extent include ICTs. It is also possible that there is a need to differentiate equestrians' learning repertoires online and offline. For example, previous research shows that equestrians are more hesitant to use SNS as a source of information on horses' injuries and diseases compared to more general information about horses and riding (Broms et al., 2021). To further investigate equestrians' (online) learning repertoires and what defines their choice of sources for horse-related knowledge, we will discuss results on what type of information sources equestrians trust in the following section.

### Agency and Trust

As mentioned previously, the interviewees underline that it is important for them to have someone around them that they can ask for advice, preferably a friend with more experience, an educated trainer, or a veterinarian. Having other, more knowledgeable and experienced equestrians in the nearby environment (*In Real Life)* gives a feeling of having a “safety net,” as several of the participants put it. Here, two interviewees discuss the matter:

R2: I think it's important to have coaches around you.
R1: Yes, but when I lived at my father's place, I had a long distance to a trainer and my mother, so then I thought it was really good with the internet, but here it's another thing. [she is currently a student at an equestrian upper secondary school.]
R2: Yes, here we have coaches to ask all the time and then you don't need to look for answers on the internet. But if you are located at a place without a coach, it can be really good to look for advice on the internet.

The importance of having people to ask is also underlined in another interview:

R1: Not everyone has knowledgeable horse people around them. They live out in the woods and have their parents or someone else and do not know a lot. They may not have so many people around them and then social media becomes the thing.

The quotations show that it is important for equestrians to have sources of knowledge available around them. However, not any kind of sources will do; they have to be sources that they trust. The question here is, who do equestrians trust and why? Our interviewees explain that they would rather have access to sources of knowledge through persons in their nearby environment than use ICTs to obtain the information. However, they are convinced that other equestrians use ICTs as a platform for knowledge exchange. Some of the participants express how they have used ICTs when they did not have access to someone to ask at the time (for example, a friend in the stable). These results are in line with previous studies showing that ICTs are not the most preferred information source for equestrians (Hockenhull and Creighton, 2013; cf. Bolwell et al., 2013; Lofgren et al., 2016). This study, together with previous research, show that equestrians generally trust and prefer to collect information from authoritative horse-people, such as veterinarians, trainers, and more experienced equestrians. However, as mentioned by the interviewee above, not everyone has this support in their immediate surroundings.

Many of the participants in this study express that they have the ability to critically evaluate and assess information. As with many of the respondents in the questionnaire, the interviewees in the quotes above express that they are rather skeptical, but at the same time, they do seem to use ICTs to obtain information about horses and riding, at least to some extent. Most of the participants are rather confident and aware of the need for critical assessment when it comes to obtaining information through ICTs. Accordingly, many of them can be described as either confident or skeptical evaluators. We see that they hold a high degree of agency or, at least, they believe so themselves. On the other hand, there are participants who show naïve characteristics, at least to some extent. For example, two interviewees discuss that they do not turn to ICTs (more specifically SNS) to obtain knowledge, but sometimes they just stumble upon something useful without really realizing it at the time. To further analyze critical assessment behavior among equestrians, we believe that it is necessary to create increased understanding of what agency means for equestrians. Here, it is interesting to mention the Dunning-Kruger effect, as critical assessment is a concept most of the equestrians seem to be very confident in. In addition, we question if they are overestimating their abilities to critically assess information distributed on ICTs. We will discuss this further below.

In general, the participants have low trust in other equestrians' capabilities to critically assess information. These “other groups” are often mentioned as less experienced, too young, or too old to have the capability to be source critical enough. Many interviewees say that they would not use social media to obtain information about, for example, injured horses themselves. However, they are certain that “other riders” do so. Namely, these “other riders” are claimed to have a lack of agency and are used or seen as “strawmen” by other groups of equestrians. Below, a group of interviewees discusses who these “other equestrians” are:

Researcher: Are there many riders who turn to social media when they, for example, have an injured horse?
R1: Yes, many do.
R2: It's like very accessible to do so…and you get quick responses.
Researcher: Who do you think are doing it?
R2: I think its people who don't have that much knowledge about horses.
R3: Yes, it's often like rich parents who have a child that likes horses, so they buy a horse, and if they're not knowledgeable and realize that the horse is lame or sick, they don't know better or aren't educated enough to know that the best thing to do is to call a vet. And then they go for the simple solution and google the symptom to see what it could be.
R4: Yes, there's many people who act like that.
R2: But it can have an economical aspect as well. There are those who don't want to call a vet because it's expensive, and then they ask people if they have been through a similar situation with their horse and ask if they think you should call the vet.

The interviewees are convinced that one needs a certain amount of experience with horses to be able to use ICTs to obtain and exchange knowledge about horses and riding in a safe way. Therefore, we argue that equestrian agency involves, above all, practical experience with horses. However, agency likely also includes horse-related education, but this is not something that the participants mention in this study. Imagined affordances is, as presented above, a notion explaining how people shape their media environments, perceive them, and have agency within them (Nagy and Neff, 2015). Here, we clearly see that practical experience is a part of equestrians' imagined affordances. The interviewees also mention that equestrians who cannot afford, for example, veterinary fees, might consult other (online) sources instead. In other words, there might be a risk that horse-owners or riders with limited economic recourses might have no other choice than consulting ICTs in some situations. Does this mean that ICTs are more important than knowledge recourses for those with limited economic recourses? This question needs to be further investigated because this is not something that we have explicitly asked about. Further, the interviewees seem quite confident that they possess the right amount of experience (i.e., they have equestrian agency) themselves. Based on the qualitative results, it is therefore interesting to see whether there is any correlation between equestrians' use of ICTs and SNS and their age and horse-related experience. The quantitative results are shown in Tables 2, 3 below, introduced by a description of the main results.

**Table 2:** Characteristics of Swedish and Norwegian riders' use of ICTs in relation to age.

**Characteristics**	**Age low** **(*n =* 129)** ***n* (%)**	**Age medium** **(*n =* 288)** ***n* (%)**	**Age high** **(*n =* 1,042)** ***n* (%)**	**All** **(1,459)** ***n* (%)**	***P*-value**
Use SNS in general					0.061
Yes	129 (100)	284 (98.6)	1,012 (97.1)	1,425 (97.7)	
No	0 (0)	4 (1.4)	30 (2.9)	34 (2.3)	
Use SNS to obtain horse-knowledge^*^					0.042
Yes	70 (66.7)	136 (56.9)	482 (54)	688 (55.6)	
No	35 (33.3)	103 (43.1)	411 (46.0)	549 (44.4)	
Use ICTs to obtain horse-knowledge^**^					0.465
Yes	89 (85.6)	215 (90.0)	798 (89.3)	1.102 (89.1)	
No	15 (14.4)	24 (10.0)	96 (10.7)	135 (10.9)	

*Note: Confidence intervals (95% CI). p ≤ 0.05*.

* *Missing values = 222*.

** *Missing values = 222*.

**Table 3:** Characteristics of Swedish and Norwegian riders' use of ICTs in relation to horse experience.

**Characteristics**	**Experience low** **(*n =* 35)** ***n* (%)**	**Experience medium** **(*n =* 317) *n* (%)**	**Experience high** **(*n =* 1,107) *n* (%)**	**All** **(1,459)** ***n* (%)**	***P*-value**
Use SNS in general					0.182
Yes	34 (97.1)	354 (99.2)	1,077 (97.3)	1,425 (97.7)	
No	1 (2.9)	3 (0.9)	30 (2.7)	34 (2.3)	
Use SNS to obtain horse-knowledge^*^					<0.001
Yes	21 (80.8)	170 (64.9)	497 (52.4)	688 (55.6)	
No	5 (19.2)	92 (35.1)	452 (47.6)	549 (44.4)	
Use the internet to obtain horse-knowledge^**^					0.464
Yes	22 (84.6)	239 (90.9)	841 (88.7)	1,102 (89.1)	
No	4 (15.4)	30 (10.1)	113 (11.4)	148 (11.3)	

*Note: Confidence intervals (95% CI). p ≤ 0.05*.

* *Missing values = 222*.

** *Missing values = 222*.

Table 2 shows that 97.7% of all respondents (*N* = 1,459) use SNS in general, and 55.6% report that they use SNS to obtain knowledge about horses and riding. Table 2 also shows that it is more common to use ICTs rather than SNS to specifically obtain horse-related knowledge. There is a difference in relation to age (low, medium, and high) in the responses to the question “Use of SNS to obtain horse-knowledge” (chi-square = 6.329, df = 2, *p* = 0.042). This means that there is a difference between the use of SNS as a source of knowledge between the different age groups, where the younger equestrians use SNS to a higher degree than the older. However, the difference is weak since phi is <0.1 (phi = 0.129). Notably, no significant difference was found relating to the use of ICTs as a source of knowledge between the age groups.

Table 3 shows that there is no significant difference in relation to horse experience and use of SNS in general. However, there was a significant difference between the three experience levels in the responses to the question “Use SNS to obtain horse-knowledge” (chi-square = 19.833, df = 2, *p* = <0.001). However, this correlation is low (phi = 0.072). Compared to the medium-experience and high-experience groups, a higher percentage of respondents in the low-experience group has responded that they use SNS to obtain knowledge. This result is in line with the interviewees' claim that it is mostly less-experienced equestrians who use SNS to specifically obtain information about horses and riding.

A comparison between Tables 2, 3 shows that 80.8% of respondents with low experience report that they use SNS to obtain knowledge about horses and riding, and 66.7% of respondents in the low-age group do the same. These results indicate that riders with a low degree of experience are more likely to turn to SNS to obtain knowledge about horses and riding than riders in the youngest age group. Furthermore, a comparison between the two tables shows similar-to-identical results between the age groups and experience groups in terms of the use of ICTs as a source of knowledge. Thus, the results from both the quantitative and qualitative study underline that equestrians with less experience are more likely to turn to SNS to obtain information about horses and riding. As we measure experience according to how many years the respondent has had riding and dealing with horses, many respondents in the low-experience group also inevitably belong to the low-age group. This means that the online repertoires for the younger age groups may be similar to those for inexperienced riders. Thus, it is likely that respondents in both groups will keep on using ICTs to obtain information about horses and riding when they get older and more experienced. These results are in line with Bolwell et al.'s (2013) argument about age demographics in relation to the use of SNS as knowledge platforms, where most of the younger respondents prefer to use SNS.

### Accessibility and Trust

To further deepen our understanding of equestrians' (online) learning repertoires, the respondents were asked whether there was a lack of information about horses and riding on ICTs. Out of 1,129 responses, only 324 (28.7%) answered “yes.” In other words, most of them were content with the information they could find online. In addition, the respondents who answered “yes” were asked to describe the type of information they were lacking. A few of the respondents noted that they did not know what type of information they were lacking. An analysis of the responses to the aforementioned question revealed two themes: accessibility and trust. Since the answers were rich in content and give an in-depth understanding of the type of information equestrians are missing on ICTs, each theme is represented in the quotes shown in Tables 4, 5 below. Table 4 shows quotes representing the first theme, accessibility, which is divided into two categories: “access to simplified information” and “access to reliable information.”

**Table 4:** The theme accessibility.

**Accessibility**	
Access to simplified information	There is certainly a lot that could be simplified and accessible through apps etc.
	There is a page missing where you can find “everything.”
Access to reliable information	I'm lacking access to new research and discoveries that are easily accessible from reliable sources. Not only things that someone “thinks” or “has experience in” but something that there is actually evidence for!
	There is a lot, but it is not available to everyone, such as scientific studies.

**Table 5:** The theme trust.

**Trust**	
Mistrust (How is this true?)	I would like an 1,177^*^ for horses. Information that one can trust.
	Horse feed, all sites are connected to brands that want to sell a product. I wish for an impartial site.
	Above all, the scientific and evidence-based perspective. *HästSverige* is good, but there is so much information that is based on personal opinion online, and advice that is not correct at all. It's scary when people take advice on horse health from anyone and do not consult their veterinarian first.
Distrust (How is this false?)	There is a lot of information, but a large part is contradictory or not accurate.
	Do not know, but far too many ignorant people without either common sense or horse experience spread their incorrect knowledge, and people believe them. Everything from theories about feed, hoof care, wound care, and general care of the horse to riding training and handling!
	Most of it is rubbish, loose opinions, or advice that is based on someone's experience of their own horse. It is difficult to assess whether a particular site is serious and of high quality.

* *1,177 is the Swedish emergency/medical call center*.

As presented in Table 4, the results show that the respondents want accessible information about horses and riding, but not any kind of information. They seek reliable information that is accessible to everyone. The questionnaire shows that, rather than pointing to specific information, several of the respondents emphasize the importance of knowing whether the information found on ICTs can be verified. Evidently, there are equestrians who are interested in using ICTs to obtain knowledge about horses. However, there are others who are critical of information shared on ICTs and underline that no more sites are needed because horsemanship, riding, and the work around the stable cannot be taught on the internet at all. Table 5 shows responses in relation to the second theme, trust, and just like Table 4 above, this theme is divided into two categories. The two categories are based on Haider and Sundin's (2020) theoretical implications around mistrust and distrust.

Many participants in this study express that they are lacking research- or evidence-based information about horses and riding on ICTs. These respondents stress that there is too much unverified information and information based on what people think and like that are available on ICTs. Table 5 shows responses related to both mistrust and distrust. The responses related to mistrust express a clear need for trustworthy sources online. On the other hand, the responses in the category distrust showcase some respondents' belief that most information online is rubbish or simply not accurate. We argue that this lack of trust is part of the equestrian community's imagined affordances.

According to the equestrians in our study, it is particularly problematic to have non-trustworthy information about horse injuries or diseases circulating online. They stress that this may negatively impact horse welfare. Further, the respondents expressed that they seek information on how to set up training plans and more in-depth information on how to train horses and riders created by educated coaches. Some of the participants point to how difficult it can be to know whether certain information on, for example, horse feed and equipment can be trusted as there are commercial agents on the internet spreading information about their products. Just like in the quantitative data, the focus group participants express that there is a clear need for a platform or even several platforms where you can find trustworthy information about horses and riding through ICTs. The participants in both the quantitative and qualitative study say that they want quick access to information that they can rely on—perhaps a kind of horse Wikipedia (as the interviewees express below) or 1,177 (as expressed in Table 5 above). This is how a couple of equestrians respond during a focus group interview when they are asked whether they are missing something regarding information about horses and riding online:

Researcher: Is there anything missing in your opinion?
R4: Yes, that would be a page that is based on facts that you could really trust.
R3: It should be a page like a horse Wikipedia where it says who has been writing it and when.

Another respondent addresses the need for a place online where equestrians can ask questions and where only true experts can respond:

R2: But the question that you had before, whether there is something lacking, perhaps a safe zone is needed. I imagine a webpage where one could ask questions to experts who answer on their time off or something. But it's only experts that can respond to the questions. Because then you perhaps only get like three responses instead of three hundred.

The results in this article show that equestrians' individual factors of online repertoires are defined by curiosity (to take part in others' experience), an eagerness to learn new things about horses and riding (to extend the knowledge about horses and riding), and availability (because information online is easily accessible and, in many cases, a quicker way to retrieve information than, for instance, by reading a book). Furthermore, trust, or perhaps the lack of trust toward online sources, is central when mapping equestrians' (online) learning repertoires. The lack of trust toward the available online sources' indicates that the structural factors (i.e., the types and quality of online sources available to specific users) is a pressing issue in the equestrian community. The equestrians express an increased need for trustworthy, reliable, and accessible sources of information online.

## Concluding Discussion

By analyzing equestrians' online repertoires (outcomes of structural, positional, and individual factors), this article contributes with increased knowledge and understanding of equestrians' online habits. First, this study shows that the participants are generally not satisfied with the availability and the quality of horse-related online content. We argue that the underdeveloped *structural factors* in relation to horse-related information online leads equestrians to turn to *search engines* (such as Google), *SNS* (such as Facebook and Instagram), and *commercial websites* instead of *official websites* and *research*. Second, this article shows that horse experience is the most important *positional factor* in the equestrian community. Horse experience seems to influence everyday online repertoires among equestrians. That is, the results reveal that online repertoires differ between equestrians with different levels of horse experience. Riders with less experience turn to *SNS* to a higher extent than riders with more experience. Third, the *individual factors* of online repertoires are defined by curiosity, eagerness to learn new things, and availability of information. The qualitative study also shows that economic conditions may play a role in relation to *individual factors*. We argue that the increased knowledge of equestrians' online repertoires may support the equestrian community to further understand in what way equestrians negotiate and renegotiate the meanings of technology. According to Nagy and Neff (2015) imagined affordances helps us to understand the complex nature of the relationship between humans and technology and offers a framework for studying the gap between users' experience of technologies and the features or qualities of the technology. As discussed above, there are studies on how equestrians in different countries and contexts search for, and prioritize knowledge about horses and riding obtained online (Bolwell et al., 2013; Byström et al., 2015; Lofgren et al., 2016; Dashper, 2017; Hii et al., 2020; Broms et al., 2021; Radmann et al., 2021), but there are no studies on how equestrians navigate the cultural and social aspects in the equestrian community on the one hand and the features and qualities offered to equestrians, on the other hand, through technology. With our results on equestrians' online repertoires and further discussion in connection to imagined affordances, we argue that we have taken a first stance toward new ways of understanding in what way equestrians shape their media environments, perceive them, and have agency within them.

In addition, the results indicate that equestrians find the ability to assess information as an important yet challenging task. However, here, we see the results pointing in different directions. On the one hand, many equestrians clearly express that they would rather stay away from obtaining information about horses and riding on ICTs. On the other hand, the data, together with previous research, indicates that many equestrians see ICTs as important platforms for discussing and exchanging information about horses and riding (cf. Byström et al., 2015; Dashper, 2017; Broms et al., 2021; Radmann et al., 2021). Many participants in this study see ICTs as an ideal place to get inspiration from other riders and to stay up to date with what is going on in the equestrian world. At the same time, they express that they want to stay away from using it as a space for knowledge exchange. This is because they see ICTs as an unsafe space where too much false information is produced and shared by other, mostly more inexperienced, equestrians. The results in this study show that the term *(online) learning repertoires* is appropriate when discussing the relationship (or sometimes, rather, *clash*) between the traditional culture in equestrian sport and the contemporary media user. The brackets around the word *online* represents how the equestrians in this study clearly express that their learning repertoires do not only contain online sources.

We suggest that the notion of (online) learning repertoires help us deepen the understanding about the complex relationship between equestrians and ICTs as knowledge platforms. As previously mentioned, equestrian online repertoires are currently defined by unsatisfying quality and availability, the influence of horse experience, and an eagerness to obtain new information about horses and riding. Here, the abovementioned complexity around imagined affordances and, ultimately, the space between the users' (i.e., equestrians') perception and reception of technologies becomes visible. The interviewees' expressed eagerness to learn new things about horses and riding clashes with their arguments concerning the lack of available and reliable sources of information online. To further understand this complex issue, we have analyzed the types of knowledge sources that are prioritized and trusted among equestrians.

The results show that accessibility, agency, and trust are key terms when mapping equestrians' preferred knowledge platforms. Further, in line with Haider and Sundin's (2020) study, the confident and skeptical evaluators were positioned as ideals, something to strive for, whereas the naïve and non-evaluators are examples of what others do, something from which to keep distance. In general, the participants in this study see themselves as confident evaluators with a high degree of agency (i.e., experience with horses). In contrast, they see other groups of equestrians, specifically more inexperienced riders, as naïve or non-evaluators incapable of assessing information obtained through ICTs. These results indicate that it may be stigmatizing to openly communicate that one goes online to gain knowledge about equestrianism, at least if one is not that experienced with horses. Thus, it may be more suitable to put the brackets around *learning* so the expression is *online (learning) repertoires*, showing that equestrians only sometimes go online to obtain information about horse and riding. Concurrently, findings in both the qualitative and quantitative data show that equestrians with low experience are more likely to use SNS to obtain horse-related knowledge, something that could be understood by using the Dunning-Kruger effect. However, due to a low response rate among equestrians with low experience in this study, further research is needed to strengthen the knowledge on the relation between lack of experience and the use of SNS to obtain knowledge on horses and riding. As previously stated, the inexperienced equestrians in our study are mainly in the low-age group (i.e., young).

The qualitative study supports the quantitative results. Namely, the interviewees argue that riders who lack experience and support from other more experienced equestrians are more likely to turn to ICTs and, more specifically, SNS to obtain information about equestrianism. The interviewees also point out that less experienced riders and riders without the economical recourses to consult a professional are more likely to consult with online sources such as Facebook groups. Thus, we conclude from the results that the focus around knowledge exchange and the development of information within the equestrian community should be on experience and recourses (or rather lack thereof) rather than age. We argue that simplified, accessible, and reliable information on horses and riding available through ICTs can prevent the decrease of quality care for horses, of which the equine industry has been warning about.

Further, we argue that equestrians' hesitance in using ICTs as a source of knowledge, their confidence in being capable of critically assessing information obtained through ICTs, and their view on other equestrians lack of capability in critically assessing information obtained through ICTs are all things that permeate contemporary stable culture(s). According to Haider and Sundin (2020), the shift from mistrust to distrust, in terms of critical assessment behavior, leads to a decreased trust in institutions and the media. However, the results in this article raise the question of whether the distrust is directed toward equestrians outside of ones nearby environment rather than toward institutions. Finally, our findings in relation to (online) learning repertoires or online (learning) repertoires among equestrians have the following implications: Is it not better for institutions to work with ICTs instead of being critical and asking riders to refrain from using them? In addition, if ICTs are not seen as trustworthy, our results indicate a riskier situation where a lack of trustworthy online platforms for information exchange on equestrianism might lead equestrians increasingly to turn to friends in the stable. Is that better than turning to reliable online sources? We argue that these questions need further focus in coming studies.

As concluding remarks, todays' online media landscape facilitates communication on how sports practitioners can develop in their sport. In addition, there is a need for sports and educational institutions to see the increased role of the individual as “a facilitator of knowledge” through ICTs. Even if equestrian sport is unique in terms of the human-horse relationship, where the human must always have the horse's welfare in mind, our study shows that ICTs play a role in the development and exchange of knowledge within sports in a wider perspective. Therefore, we suggest further studies within sports sciences to focus on the relationship between the construction, mediation, and materialization of power and social relationships taking place through ICTs.

## Data Availability Statement

The original contributions presented in the study are included in the article/supplementary material, further inquiries can be directed to the corresponding author/s.

## Ethics Statement

Ethical review and approval was not required for the study on human participants in accordance with the local legislation and institutional requirements. The patients/participants provided their written informed consent to participate in this study.

## Author Contributions

LB, KB, AR, and SH contributed to the conception and design of the study. LB, AR, and SH collected the material. LB performed the initial qualitative analysis and wrote the first draft of the article. KB performed the initial quantitative analysis and wrote sections of the manuscript. All authors contributed to the final analysis of the data, manuscript revisions, and approved the submitted version.

## Funding

This work was supported by the Swedish-Norwegian Foundation for Equine Research (grant number: H-17-47-290).

## Conflict of Interest

The authors declare that the research was conducted in the absence of any commercial or financial relationships that could be construed as a potential conflict of interest.

## Publisher's Note

All claims expressed in this article are solely those of the authors and do not necessarily represent those of their affiliated organizations, or those of the publisher, the editors and the reviewers. Any product that may be evaluated in this article, or claim that may be made by its manufacturer, is not guaranteed or endorsed by the publisher.
